# President’s Address to the Mississippi Valley Association, March 6, 1867

**Published:** 1867-06

**Authors:** G. W. Keely


### PRESIDENT'S ADDRESS TO THE MISSISSIPPI VALLEY ASSOCIATION, MARCH 6, 1867.

BY G. W. KEELY, D.D.S.

Gentlemen:—That Creator, to whom we should, all, with "equal reverence, bow," has permitted us to meet again, to enjoy another season of social intercourse, and to contribute our mite to advance the great interest of our chosen specialty.

This Association, now the oldest Dental Society in existence, was organized in this city, in the Lecture Room of the Medical College of Ohio, on the 13th of August, 1844. Dr. James Taylor announced the object of the meeting, when Dr. Joseph Taylor, of Maysville, Ky., was called to the Chair, and Dr. W. B. Ross, of Newport, Ky., was appointed Secretary. A Constitution and By-Laws were adopted and Officers elected for the ensuing year.

There were present on this occasion, some twenty-eight gentlemen, some of whom are still earnest and active working members, who scarcely ever fail to be present at our annual meetings.

You will pardon me, gentlemen, if I mention the names of a few of the latter : Dr. James Taylor, Drs. Ullery, Rogers, Allen, Berry, Wheeler, and Goddard.

Others, who were present on this interesting occasion, whom we delight to honor, and remember as among the bright lights of our profession, have, in the course of nature, been gathered to their final homes—Ross, Hunt, Hullihen, and others—have gone to that "undiscovered country, from whose bourne no traveler returns." And thus we are reminded that, we two, are but mortal, that here we have no "continuing city," and that the tears of affection, which tremble upon the tombs of these departed brothers, will surely soon be transferred to ours.

At this, our Twenty-Third Annual Meeting, I venture the assertion, that none of us can fully appreciate the importance of this organization. Here we discuss the different subjects, most intimately connected with the interests of our profession, and by a free interchange of ideas, on the various modes of practice, we are mutually benefited, none feeling so selfish as to keep within himself any important improvement he has been fortunate enough to make, during the past year. But it is his pride to make it known for the common good.

A Committee report annually on all important improvements, and mechanical appliances, which alone will richly compensate any one who has at heart the interest of his patients or the advancement of his profession.

I appeal to you, gentlemen, who have been attending these meetings for the past twenty-three years, if you can afford to stay away, when it is possible to be present. If you will not be the loser by so doing.

How often we hear it said, "The more I attend Dental Societies, the more I wish to." Why? Simply because we are improved, our hearts grow larger, our affections are drawn out, and we are made to love our specialty, and our professional brethern more than ever before. It is said, that in "union there is strength." You, gentlemen, will realize this fact, on your return to your homes, feeling stronger, and more competent than ever before, to battle with the difficulties you meet almost daily in your practice.

This being a fact (acknowledged by all society men,) is it not our duty to put forth every honorable effort, to induce our professional brethern to become members of some Dental Society.

For ten long years, I urged upon a friend, the importance of becoming a member of this Society. His frequent *excuse* was so common, I need hardly mention it. "Press of business precluded the possibility of his absence from his office, even for two or three days."

I persevered, and finally came out conqueror. After his return from the first meeting, to his much loved office, I was the recipient of a long and interesting letter, from which I was convinced that my persistent efforts were fully appreciated. He said, "I never entertained the remotest idea, that it was possible for any one to learn so much in so short a time."

Our Society is not sectional, for her affectionate arms extend to almost every State in the Union, and even to other countries. Her children are numbered by the hundred, and many of them are among the brightest ornaments of our profession.

Gentlemen, of the Mississippi Valley Dental Association, we need not urge upon you, on this occasion, the importance of a thorough Dental education. This magnificent Temple, "The Ohio College of Dental Surgery," in which we meet to-day—the only one in the world, erected and owned by a Dental Association, for the sole purpose of Dental education —speaks to us in tones not to be misunderstood. You who are familiar with the course of study pursued in this Institution, can form some idea in regard to what it is doing to elevate our profession.

Other Dental Colleges are doing a noble work. At Baltimore, Philadelphia, New York and St. Louis, the good work goes bravely on.

We should all be impressed with the idea of looking much to individual effort, in elevating our chosen profession. We need have little hope of being protected by the laws of the land—efforts in this direction have signally failed in the Medical Profession, and may in ours.

Let it be the resolve of every Dentist, not to take a student unless he is possessed of a good English education, and one whom he would feel proud to introduce to his family and friends, with a pupilage, not less than two years, and requiring him to attend a full course of Lectures in some Dental College, and in whose *integrity* he has the most *implicit confidence.*

Gentlemen, let us insist upon a thorough education, making the standard as high as possible, and thus make the line of separation between the truly educated Dentist, and the pretender, so distinct, that the blind may know how to choose between them. And the "dear people" too, are to be educated up to the point of appreciating good operations, and to know whom to trust.

No intelligent patient will pass from the hands of a good operator to an empiric without discovering the fraud. But should he unfortunately call upon the latter named, first, he will surely soon realize, when perhaps too late, that he has been victimized.

But I have detained you too long, let our efforts in the future be more earnest, than ever before, for the elevation of our specialty.

In conclusion, gentlemen, let us hope that the day is not far distant, when the uneducated Dentist, and the one who fails to take an active interest in Dental Associations, will utterly fail in commanding intelligent patients.

